# Genome Wide Assessment of Young Onset Parkinson’s Disease from Finland

**DOI:** 10.1371/journal.pone.0041859

**Published:** 2012-07-24

**Authors:** Dena G. Hernandez, Michael A. Nalls, Pauli Ylikotila, Margaux Keller, John A. Hardy, Kari Majamaa, Andrew B. Singleton

**Affiliations:** 1 Laboratory of Neurogenetics, National Institute on Aging, National Institutes of Health, Bethesda, Maryland, United States of America; 2 Department of Neurology, Turku University Hospital, Turku, Finland; 3 Reta Lilla Weston Laboratories and Departments of Molecular Neuroscience, UCL Institute of Neurology, Queen Square, London, United Kingdom; 4 Institute of Clinical Medicine, Department of Neurology, University of Oulu, Oulu, Finland; Oslo University Hospital, Norway

## Abstract

In the current study we undertook a series of experiments to test the hypothesis that a monogenic cause of disease may be detectable within a cohort of Finnish young onset Parkinson’s disease patients. In the first instance we performed standard genome wide association analyses, and subsequent risk profile analysis. In addition we performed a series of analyses that involved testing measures of global relatedness within the cases compared to controls, searching for excess homozygosity in the cases, and examining the cases for signs of excess local genomic relatedness using a sliding window approach. This work suggested that the previously identified common, low risk alleles, and the risk models associated with these alleles, were generalizable to the Finnish Parkinson’s disease population. However, we found no evidence that would suggest a single common high penetrance mutation exists in this cohort of young onset patients.

## Introduction

Over the past 15 years the genetic investigation of Parkinson’s disease has successfully identified many disease-causing mutations, and these have been used extensively to understand the etiology of this complex disease. Genome wide association (GWA) studies have been applied to explicitly test the common disease common variant hypothesis in PD [1]. This has led to the identification of a significant number of novel risk loci [2]–[8]. From an etiologic perspective it is notable that some of these loci include genes known to contain mutations that cause neurodegenerative diseases [9].

Within isolated populations, allelic and genetic heterogeneity tends to be reduced and as a result single founder mutations can be quite common in the disease population. Thus the application of GWA in these populations can also lead to the resolution of loci containing highly penetrant mutations, particularly in sub-types of disease that have a substantial monogenic component within conserved populations [10], [11]. Perhaps the best example of this is the description of association at chromosome 9p in a GWA of familial ALS cases from Finland. This ultimately led to the identification of the pathogenic expansion within *C9orf72*, which causes a substantial number of ALS and frontotemporal dementia cases worldwide [12], [13].

We embarked upon a series of experiments aimed at understanding whether a similar monogenic component may be detectible in PD in the same population. To test this idea we used high content SNP genotyping in a series of young-onset (<55 years of age) Parkinson’s disease cases from Finland. This group was used because the Finnish ancestry should reduce allelic and genetic heterogeneity when compared to a more outbred population [14], [15], and because substantive evidence exists suggesting an enrichment of monogenic disease in earlier onset PD [16].

## Results

**Figure 1:**
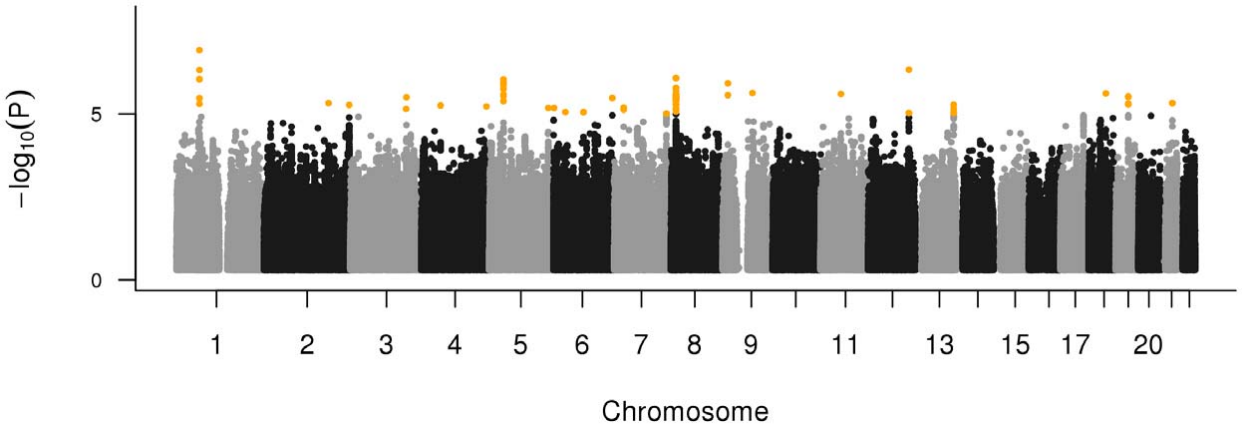
Manhattan plot showing results of GWA testing between 378 PD cases and 496 controls from Finland, genomic inflation factor  = 1.0659.

The primary dataset analyzed included genotype data on 387 PD patients with an age at first treatment of less than 55 years and 496 controls from the Vantaa 85+ Study. Standard GWA procedures were instituted (see materials and methods) to identify putative loci associated with young-onset PD, because age and gender adjustments did not significantly affect overall results and model fit, we opted to utilize the most parsimonious model, excluding these covariates. Logistic regression models using non-integer genotype dosages adjusting for component vectors one and two from multi-dimensional scaling were tested. This was performed across a total of 5,854,841 SNPs imputed based on the 1000 Genomes Project haplotypes released in August 2009 (for compatibility with previously published IPDGC studies) passing quality control measures described below. No SNPs successfully passed multiple test correction in the GWAS phase of analyses (p-values <5E-08, summarized in Figure 1).

Next we examined SNPs previously linked to PD risk [6], [7]. Of these 18 variants, 6 were genotyped successfully and another 7 were imputed with sufficient quality in the present study (note, due to low coverage genotyping, 5 loci did not contain valid proxies that were successfully imputed at an r^2^>0.8). Although the current study was not designed to detect common low risk variants, and our effective power to do so was zero [17], it is notable that the size and direction of effect for each of these variants is consistent with the previous report (Table 1) [6].

**Table 1:** Results of association testing at published loci. OR published is based on replication phase odds ratio previously published for these SNPs [6], [7].

SNP	Minor Allele < Major Allele	MAF	IQ	Previously published OR	OR	Lower 95% CI	Upper 95% CI	P-value	Gene	CHR	BP
chr1:154105678	T<C	0.13	0.47	1.44	1.79	1.17	2.74	7.14E-03	SYT11	1	154105678
rs708723	C<T	0.40	0.99	0.86	0.93	0.77	1.13	4.81E-01	RAB7L1/PARK16	1	204005889
rs2102808	T<G	0.12	0.92	1.12	1.04	0.77	1.42	7.89E-01	STK39	2	168825271
rs34016896	T<C	0.33	1.00	1.08	1.10	0.90	1.35	3.71E-01	NMD3	3	162475558
rs11711441	A<G	0.12	0.99	0.87	0.80	0.58	1.09	1.47E-01	MCCC1/LAMP3	3	184303969
rs11724635	C<A	0.41	0.88	0.87	0.81	0.66	1.00	5.18E-02	BST1	4	15346199
rs6812193	T<C	0.33	1.00	0.91	0.86	0.69	1.06	1.48E-01	STBD1	4	77418010
rs356219	G<A	0.41	0.81	1.27	1.37	1.11	1.70	3.97E-03	SNCA	4	90856624
rs156429	C<T	0.37	0.98	0.89	0.83	0.67	1.02	7.50E-02	GPNMB	7	23272545
rs591323	A<G	0.33	0.83	0.88	1.01	0.81	1.26	9.25E-01	FGF20	8	16741462
chr8:89442157	T<C	0.03	0.63	1.29	1.04	0.52	2.08	9.13E-01	MMP16	8	89442157
rs1491942	G<C	0.14	1.00	1.30	1.14	0.85	1.54	3.67E-01	LRRK2	12	38907075
rs4889603	G<A	0.43	0.83	1.15	1.33	1.07	1.64	8.99E-03	STX1B	16	30889726

Note gene is the best proximal candidate or closest gene to the locus and may not be the true pathologically important species. Notably the power to detect association at these loci, based on previously published effect sizes, p<5E-8, and an additive model is effectively 0 (based on methodology of [17]). MAF, Minor Allele Frequency; OR, odds ratio; CI, confidence interval; IQ, Imputation quality from MACH (RSQR metric).

Likewise, using these 13 variants, risk profile analysis revealed an association between risk alleles previously defined in the IPDGC meta-analysis and disease in this Finnish cohort. This was performed using logistic regression to evaluate the effect of the cumulative risk allele dosages, p = 2.03×10^−8^ [6]. The magnitude of effects across risk quintiles was similar to those previously observed, showing a >3 fold increase in risk of PD in the highest versus lowest risk quintiles (Table 2).

**Table 2:** Summary of risk-profile analysis in the Finnish, compared to the same risk profile analysis previously performed in a series of European ancestry PD patients [6].

	P value	AUC	Risk quintile: OR (95% CI)				
			First	Second	Third	Fourth	Fifth
**IPDGC**	<2×10−16	0.63	1.00	1.43(1.27–1.62)	1.77(1.55–1.99)	2.03(1.80–2.32)	2.51(2.23–2.83)
**Finnish**	2.03E-08	0.614	1.00	1.43(0.92–2.22)	1.42(0.91–2.20)	2.431.58–3.77)	3.02(1.96–4.69)

AUC: area under the curve, indicated by the *c* index from receiver operator curves. OR: odds ratio.

Excess shared ancestry defined by identity by state (IBS) and excess homozygosity analyses were also conducted to investigate broader genetic associations within this dataset. Prior to conducting these analyses, all genotyped SNPs were pruned for linkage disequilibrium using the methods described previously by us [18]. We did not find evidence for excess relatedness among cases compared to controls either by IBS or an over burden of extended tracts of homozygosity.

Sliding window functions were employed to estimate the local rates of identity by state (IBS) differing between cases and controls; these were analyzed in 500 kb sliding windows of the genome, each overlapping by 250 kb. In this sliding window analysis, no discrete genomic regions passed Bonferroni correction for significance. This suggests that no loci were significantly over-represented in the case-only IBS calculations compared to the same analysis performed in controls. The threshold for significance was set at a minimum unadjusted P<5E-06 based on approximately 10,580 sliding windows tested throughout the twenty-two autosomes (∼2645 MB).

Homozygosity analysis was conducted based on overlapping regions contained within runs of homozygosity using PLINK’s inherent maxT permutation testing and empirical p-values were corrected by label-swapping to exclude false positives per defined region. No results exceeded the empirically determined threshold for multiple test correction. Further, all samples were within the relatively normal range of homozygosity, with a minimum Fhat of −.012 and a maximum Fhat of 0.11 (calculated as previously described [18]).

## Discussion

In the current study we failed to identify an association signal indicative of a shared, common mutation underlying younger onset PD. Our sliding window analysis, aimed at identifying genomic regions shared by cases more commonly than controls also failed to identify any significant loci. Moreover our analyses failed to reveal an excess of homozygosity in cases, thus failing to support a role for a common recessive founder mutation in this series; however, our study possessed fairly low power to detect less frequent recessive mutations and thus we cannot exclude these as a cause of PD in this cohort [18].

While these analyses suggest that there is not a predominant single monogenic cause of disease in this group, we were able to see evidence of genetic association within this cohort, consistent with previous work in less conserved populations of European descent [6]. The current work shows that the risk profiles previously proposed by us in outbred populations of European descent are generalizable to the Finnish population (Table 2).

Given the valuable nature of this cohort, we believe that further investigation using methods designed to detect mutations in the presence of genetic and allelic heterogeneity are warranted [9].

## Materials and Methods

This research was conducted according to the principles expressed in the Declaration of Helsinki. The Ethics Committee of Turku University Hospital approved this study and all subjects provided written informed consent.

In October 2007 all patients in Finland who had become eligible for reimbursement for PD medication from 1995 to 2006, who were <55 years of age and who were alive on 1 October 2007, were identified from the Drug Reimbursement Register (Social Insurance Institute of Finland database). In each case the diagnosis fulfilled international criteria for PD. A nationwide cohort of 1090 early-onset Parkinson’s disease (EOPD) patients was identified. Following exclusion of children, recently deceased and emigrants 1077 patients remained; study material was sent to each of these subjects. In addition, the birthplace and age of each of the parents were requested. Family history of PD and related disorders (dementia, gait disorder and mental illness) were also included in the questionnaire. A total of 460 (42.7%) participants gave their acceptance for the study. All participants provided a blood sample, completed questionnaire and informed consent. All participants provided a blood sample, completed questionnaire and informed consent. Four patients reported that their PD diagnosis had been withdrawn and they were thus excluded from the study. Seven patients were excluded, because their PD diagnosis had been set at the age of 55 years. Altogether, 449 patients fulfilled the inclusion criteria. These participants recruited as cases were genotyped using the Illumina Human660W version 1 BeadChip as per the manufacturers directions (Illumina Inc., CA).

Control samples were taken from the Vantaa85+ study population. This control group comprises all persons aged 85 years or older who were living in the city of Vantaa on April 1, 1991. Of the 601 eligible subjects, peripheral blood (and DNA) samples have been obtained from 515 study subjects. The Vantaa85+ cohort was genotyped using Illumina HumanCNV370 BeadChips as per the manufacturers instructions (Illumina, Inc., CA). Basic quality control was undertaken including verification of self-reported sex by examination of X chromosome heterogeneity to estimate gender from genotype data and a minimum per sample genome-wide call rate of 95% per sample in each of the two series. Because of a failed genotyping run 56 of the attempted 447 samples failed our quality control metrics because of low genotype rate. SNPs meeting the following criteria were excluded from the case and control datasets prior to merging: <95% genotyping success rate per SNP, minor allele frequency (MAF) <0.01, Hardy-Weinberg equilibrium (HWE) p-value <1E-4 in controls and <1E-7 in cases. After merging consensus genotyped SNPs across both platforms, further quality control was undertaken including filtering for missingness in cases compared to controls p-value (from chi-squared test) <1E-5 as well as nonrandom missingness by haplotype (from chi-squared test) <1E-5. The case-control series was then merged with HapMap3 populations and clustered using multidimensional scaling to verify northern European ancestry consistent with self reported Finnish ancestry. The case-control series then underwent the calculation of pairwise identity by descent, excluding any samples sharing greater than a 0.15 proportion of alleles (pi_hat >0.15) to extract only probands from related pairs, effectively excluding all first degree relatives. An additional 4 samples were removed, 3 because of excess relatedness and 1 because it was identified as a population outlier. To generate covariates for logistic regression models, multidimensional scaling was again used to quantify genetic distances between remaining members of the case-control cohort.

Case and control data were trimmed to an overlapping set of 302,463 genotyped SNPs passing quality control measures described above. Imputation of genotypes was performed using a Markov Chain based haplotyper (MACH; version 1.0.16) with reference haplotypes derived from initial low coverage sequencing of 112 European ancestry samples in the 1000 Genomes Project (as of August, 2009) [19]. These data were imputed using a two-stage design. The first stage generated error and crossover maps as parameter estimates for imputation on a random subset of 200 samples over 100 iterations of the initial statistical model. We used these parameter estimates to generate maximum likelihood estimates of allele numbers per SNP on the basis of reference haplotypes for the datasets during the second stage of the imputation. SNPs with RSQR quality estimates of less than 0.30 as indicated by MACH and a minor allele frequency of less than 0.01 were excluded from analyses of the datasets, because imputed genotypes below this threshold are likely poor quality.

We performed genome-wide dataset analyses at every site with MACH2DAT [19]. We used non-integer allele numbers as a primary predictor of Parkinson's disease in logistic regression models to account for imputation uncertainty as previously described [6]. We used basic covariates of component vectors 1 and 2 from either principal components or multidimensional scaling analyses of the case-control cohort to identify random genomic differences between genotyped data from cases and controls, which were used to adjust statistical models for covariates accounting for possible population substructure.

The risk profile analysis was performed as previously described [6], [17], [20]. Briefly, using alleles at 13 previously identified PD risk loci, cumulative risk scores were assigned to each subject. These were then ranked based on that score, and this ranked group then divided into quintiles of risk. Risk associations per quintile were then calculated with quintile 1 (lowest risk score) as the reference group. Logistic regression models adjusting for component vectors one and two from multidimensional scaling were used to quantify risk per quintile using the lowest risk quintile group as a statistical reference.

For IBD comparisons and the estimation of large runs of homozygosity, linkage-pruned datasets of genotyped SNPs were generated to avoid confounding by large blocks of highly correlated SNPs. We excluded SNPs thought to be in high LD if they met the criteria of variance inflation factor >1.05 (corresponding to a ∼1% maximum multiple correlation coefficient with each sliding window) within any sliding window of 50 adjacent SNPs which scrolled through the genome at a rate of 5 overlapping SNPs per window for the autosomes, as sex chromosomes were excluded from all analyses described in this report. All IBS calculations were based on PLINKv1.07.

Based on this LD pruned data, PLINK was used to calculate case and control specific rates of IBS (proportional allele sharing) both genome-wide and within 500 kb sliding windows which overlapped with adjacent windows by 250 kb. Permutation testing, employing a fixed 10,000 permutations, was used to compare both genome-wide and within window rates of identity by state among cases and controls, testing the hypothesis that rates of IBS would be higher among cases compared to controls.

Runs of homozygosity (ROHs) were defined using PLINK as well, also based on the LD-pruned datasets. The criteria used for defining a run of homozygosity included at least 1 MB of consecutive homozygous calls per individual, containing at least 50 SNPs, and allowing only 2 missing SNPs and 1 heterozygote. Total percent of the genome comprised of these runs was calculated using the summed length of all of a participant’s ROHs divided by the length of the autosomal genome. The frequency of ROHs was defined as the total number of ROHs per individual. Logistic regression adjusting for year of participant birth and component vectors one and two from multidimensional scaling was used to quantify risk for genome-wide burden of ROHs and ROH frequency as predictors of PD.

Homozygosity mapping was undertaken using the previously defined ROHs. Consensus regions were extracted from this set, including loci that contained at least 3 SNPs across a minimum of 100 kb found in no less than three participants. A modified version of the maxT permutation test implemented in PLINK to test for copy-number variants was employed to assess the associations between these consensus regions and PD in our study across 50,000 permutations.
